# The Chick Embryo Chorioallantoic Membrane (CAM) Assay: A Novel Experimental Model in Dental Research

**DOI:** 10.7759/cureus.74714

**Published:** 2024-11-29

**Authors:** Borislav Dusan Caplar, Marius Mihai Togoe, Domenico Ribatti, Daniela Pop, Cosmin Sinescu, Mihai Rominu, Emanuela Lidia Petrescu, Meda Lavinia Negrutiu, Eugen Melnic, Anca Maria Cimpean

**Affiliations:** 1 Department of Prostheses Technology and Dental Materials, Faculty of Dental Medicine, Dental Research Center Using Conventional and Alternative Technologies, "Victor Babes" University of Medicine and Pharmacy, Timisoara, ROU; 2 Department of Translational Biomedicine and Neuroscience, University of Bari Medical School, Bari, ITA; 3 Department of Prostheses Technology and Dental Materials, Faculty of Dental Medicine, Dental Research Center Using Conventional and Alternative Technologies, “Victor Babes” University of Medicine and Pharmacy, Timisoara, ROU; 4 Department of Dentistry, Faculty of Dentistry, “Victor Babes” University of Medicine and Pharmacy, Timisoara, ROU; 5 Department of Pathology, Nicolae Testemitanu State University of Medicine and Pharmacy, Chișinău, MDA; 6 Department of Microscopic Morphology/Histology, “Victor Babes” University of Medicine and Pharmacy, Timisoara, ROU

**Keywords:** biomaterials, bone augmentation, chorioallantoic membrane (cam), dentistry, stem cells

## Abstract

Animal experimental models are ruled out by respecting the 3Rs (Replacement, Reduction, Refinement) rules which governed the experimental research for decades with an increased tendency to minimize as much as it is possible any pain suffering or distress that the animals might feel. The chick embryo chorioallantoic membrane (CAM) model is an alternative to other experimental models due to its superior properties compared to other animal models. The CAM is painless by itself due to the lack of innervation and has no immune cells till the 11th day of incubation. Thus, it is extensively used for implanting malignant tumors and assessing them in relation to their metastatic and angiogenic potential. Also, various biomaterials from collagen to hard scaffolds can be implanted on the CAM surface and analyzed mainly related to their property of inducing inflammation. Dental research often uses mouse or rabbit models for experimental purposes. Different surgical techniques from experimentally induced periodontal disease to experimental dental implants may cause pain and suffering to animals. Due to all these arguments, the CAM model is a quick, cheap, and reliable alternative to other animal experimental models used in dental research. Despite its usefulness as an experimental model for different applications, ranging from inflammation studies to cancer research, the CAM model is insufficiently used in dental research. Currently, about 135 studies pertaining to this issue are available in PubMed, the majority of which focus on the reactivity of CAM vessels to various materials employed in dentistry. Limited data exist about the capacity of the CAM to promote osteogenic differentiation of dental stem cells or to enhance biomaterial integration into novel tissue architectures. The present review critically analyzed the use of the CAM model as an experimental tool in dental research. We selected from PubMed all the papers having as topic the CAM in dentistry by searching based on the following keywords: " chorioallantoic membrane, dentistry" or "chorioallantoic membrane, dental ". We focused on discussing the benefits and limitations of the CAM model in dental studies and its prospective role as a preclinical instrument for the assessment of dental tissues, biomaterials, or different dentistry-related substances prior to their use for various purposes in dental clinical practice. ​​​​​​The impact of the CAM model-derived preclinical findings on clinical practice will be also stated by mentioning "pros and cons" arguments. The last part of the present paper reviewed the perspective of CAM assay used in combination with other experimental techniques such as tooth organoids and also the strengths and weaknesses of other species CAM assays recently developed in ostrich and Nile crocodile CAMs.

## Introduction and background

Abrief introduction to the chorioallantoic membrane (CAM) model 

The chick embryo CAM is recognized as a significant experimental tool for investigating both normal and pathological angiogenesis, owing to its extensive vascular network [1,2]. Furthermore, the CAM is a developing tissue that does not possess a fully developed immune system and contains mesenchymal cells with stem-like capabilities, which can differentiate based on a specific microenvironment [3,4]. In this context, the CAM serves as a dependable in vivo model for evaluating the behavior of both normal and pathological tissues [5,6], different drugs and antibodies [7,8], as well as a range of biomaterials implanted on its surface [9,10]. The absence of suitable methods to assess the influence of regulators on the angiogenic response presents a problem for researchers looking into angiogenesis. The most crucial characteristics of a perfect test are physiological relevance, technical ease of use, dependability, and easy quantifiability. Utilizing the chicken embryo's extraembryonic membrane, the CAM model provides a special blend of affordability, ease of use, and quick development, which makes it a desirable choice for angiogenesis tests. The CAM platform exhibits a considerable degree of immunotolerance, facilitating the exploration of cross-species xenografts and mammalian tissue explants, for extended periods. To administer a test substance onto the CAM, create an aperture in the eggshell and position it in delayed-release polymer pellets, gelatin sponges, or air-dried on filter discs. Two primary ways exist for accessing the CAM. One approach involves permitting the embryo to develop within the shell before creating an aperture in the shell. The alternative procedure involves incubating the embryo in a Petri dish devoid of the shell. Both techniques are categorized as minimally invasive. The 'in shell' technique requires minimal maintenance and allows embryos to be maintained in advanced developmental stages. The 'ex ovo' methodology enhances accessibility to the test site, facilitating several treatments or various test sites on a single CAM, in addition to the capacity to capture images of a broader area of the CAM. 

This study emphasizes the rationale that renders the CAM assay a compelling technique for selecting suitable biomaterials for tissue engineering applications in dentistry. The CAM model is delineated as a platform for biomaterial selection in prospective medical device development, including its advantages, limitations, implementation, essential considerations, and recommendations for successful experimentation. The CAM assay has been evaluated as an in vivo testing tool for dental adhesives and other chemical compounds utilized in dentistry, with a particular focus on irritation and cytotoxic effects. The prospective clinical applications are ultimately discussed as the future of CAM assay use in dental research.

A historical background of using CAM assay as the experimental model in dentistry

The CAM is used even less frequently as an experimental model in dental medicine research. The first paper related to the use of the CAM model in dentistry was published by Lemus et al. in 1980 in a study referring to the development of embryonic tooth buds in lizards [11]. The authors highlighted the advantages of the CAM by mentioning that “the chick chorioallantoic membrane and the semisolid-liquid culture medium supply the majority of the factors required for development of these tissues” [11]. Three years later, Greenberg et al. certified the CAM's ability to support the enamel prism equivalent formation when the cap stage tooth organs were cultured on the CAM surface [12]. CAM co-culture of quail ectoderm with lizard dental papilla was followed by the tooth bud differentiation which subsequently was able to develop teeth with dentine and incipient enamel [13]. Bone remodeling and osteoclast activity were also studied by using the CAM model, starting in 1990 [14].

The irritative potentials of dental polymer products used as restorative materials, adhesives, or temporary constructs were assessed using the HET-CAM (hen's egg test-chorioallantoic membrane) methodology starting in 1999 [15]. Based on this model, Dahl demonstrated that from all prime and bonding agents, nine of 12 primers and six of 19 bonding agents were classified as strong irritants due to their ability to coagulate the CAM’s blood in less than 100 sec [12]. Dental antiseptics are highly irritant for oral mucosa. Several studies performed on the HET-CAM model showed different levels of irritative activity of various dental antiseptics [16-18]. Dental polymer's cytotoxic effects were assessed on the CAM model [19]. Sanguinarine, a plant alkaloid that is a common component of various dental products with antibacterial, antifungal, and anti-inflammatory activities that reduce gingival inflammation and supragingival plaque formation, was first tested on the CAM about 20 years ago and showed a strong antiangiogenic potential due to its ability to suppress basal and vascular endothelial growth factor (VEGF)-induced Akt phosphorylation, while it did not produce any changes in VEGF-induced activation of ERK1/2 and PLCgamma1 [20]. In 2008, a hyaluronate-based compound (AMINOGAM) was reported to have a strong healing ability in the oral tissues after dental extractions, and its positive effects on oral tissue regeneration were strongly connected to AMINOGAM ability to induce a potent angiogenic response on the CAM through a VEGF pathway mechanism [21]. The healing of irradiated avascular mandibular bone by ultrasound therapy was proved to be based on ultrasound neoangiogenesis stimulation which was assessed on the CAM [22]. Angiogenesis stimulation is one of the main mechanisms of facilitating periodontal healing, and Kauvar et al. [23] tested for the first time the angiogenic potential of enamel matrix derivatives (EMDs) implanted on the CAM and proved the angiogenic potential of amelogenin [23]. One year later, Scheel and Hermann tested the hydroxyapatite salts on the CAM with special emphasis on their irritant potential not as an experimental tool for possible bone formation and regeneration [24]. The CAM's potential use as a good experimental model of bone regeneration was indirectly suggested several years before (1972) by three studies by Coleman and Garrison [25-27] who proved that CAM cells have a high potential for calcium trafficking through cells from both components of the CAM. By having three conditions needed for bone formation (blood supply, calcium trafficking, and oxygen), the CAM proved to be a reliable experimental model for testing mandibular bone formation and regeneration reported in subsequent studies.

Starting in 2013, human dental pulp stem cells with the ability of self-renewal and multilineage differentiation capacity have been implanted on the CAM [28]. Bronckaers et al. [28] reported human dental pulp stem cells' angiogenic properties mediated by VEGF, monocyte chemotactic protein-1 (MCP1), and endostatin. Two years later, Oliveira et al. [29] demonstrated the human dental pulp stem cells' osteoblastic differentiation potential. In 2018, Cirligeriu et al. [30] reported the CAM as a suitable platform for hyaluronic acid-induced osteoblastic differentiation by testing bone substitutes usually used in dentistry clinical practice.

**Figure 1:**
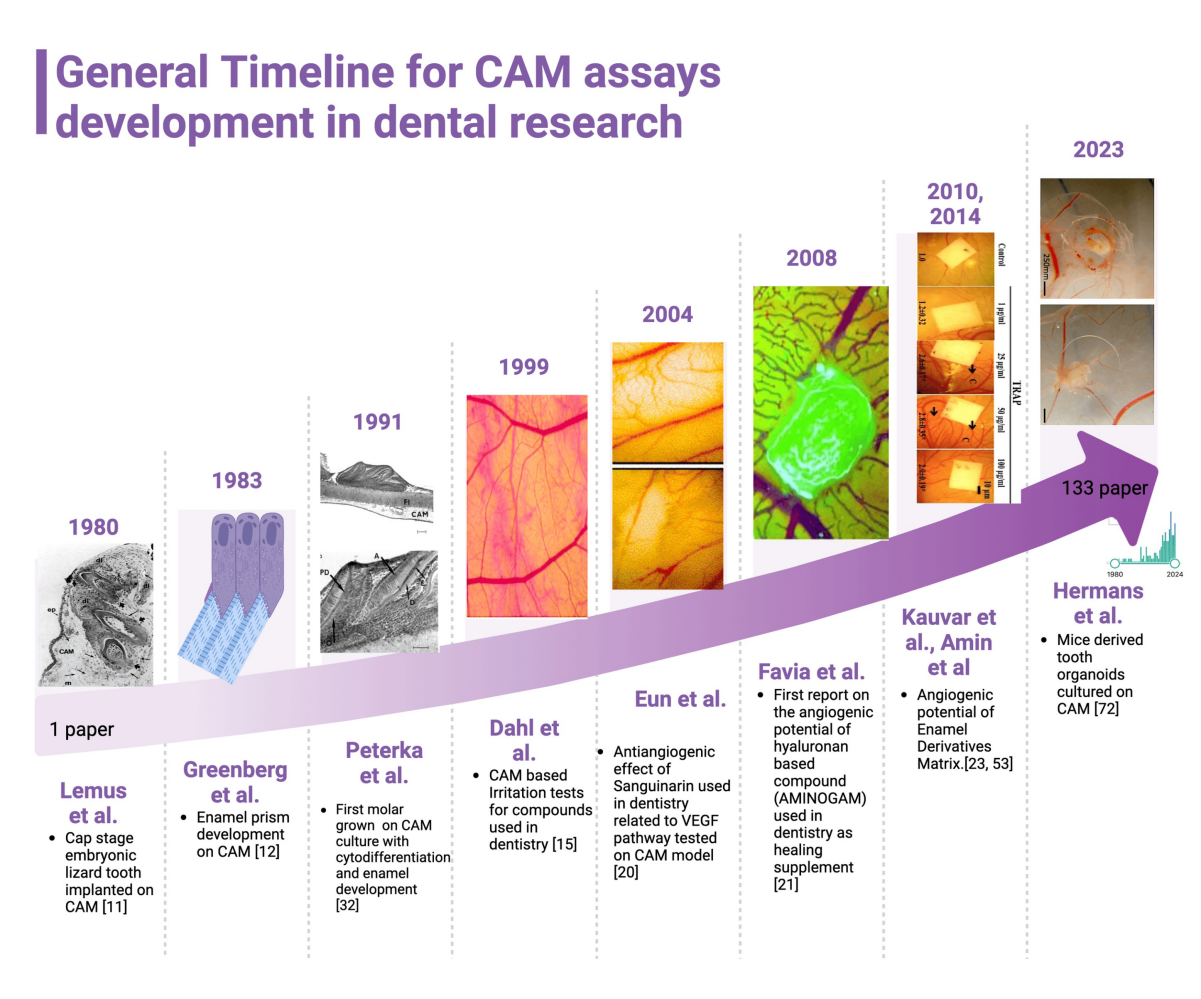
General timeline of CAM assay development in dental research during time Since 1980, CAM assay started to be used for dental research by using embryonic developing dental tissues to study tooth morphogenesis. Later, Greenberg proved the utility of CAM assay and its properties as a valuable platform for enamel differentiation. These preliminary studies were followed by CAM assay use for toxicological tests and also for the assessment of the angiogenic potential of various materials used in dentistry clinical practice. Recently, a combination of CAM assay and tooth organoids was published by Hermans et al. as an in vivo alternative to in vitro studies using organoids. This is an original figure created by the authors themselves. Figure 1 is created using special software (https://www.biorender.com/) dedicated to the graphical collage which may be inserted in the research articles. This service provides originality certificate for each created picture and also it gives us permission for publication. CAM: Chorioallantoic membrane

Together with lizards’ teeth, the unique mature tooth cultivation on the CAM was reported by Langille and Hall in 1988 for lamprey teeth grafting on the CAM for the study of dental cell migration [31]. In 1991, Peterka et al. [32] highlighted the superior potential of the CAM assay for tooth-related tissue differentiations compared to in vitro models. The authors reported that in the CAM assay, the 17-day fetuses derived teeth germs have the ability of complex cytodifferentiation compared to in vitro assays. In the CAM assay, the cytodifferentiation of tooth germs was characterized by the presence of odontoblasts, ameloblasts, predentine, dentine, and enamel while in vitro assays lacked the enamel organ and dental pulp development. A timeline of the CAM assay use and development in dental research has been summarized in Figure 1.

Other experimental models involved in ovo manipulation for harvesting tooth buds for experimental purpose are rare and in preparation. Recently, Truter and Myburg reported in ovo preparation of Nile crocodile embryos for harvesting teeth buds for future research, because Nile crocodile teeth have the ability of self-renewal during adult life [33].

## Review

Morphologic and biochemical similarities between the CAM structure, developing tooth, and mature human dental pulp

**Figure 2:**
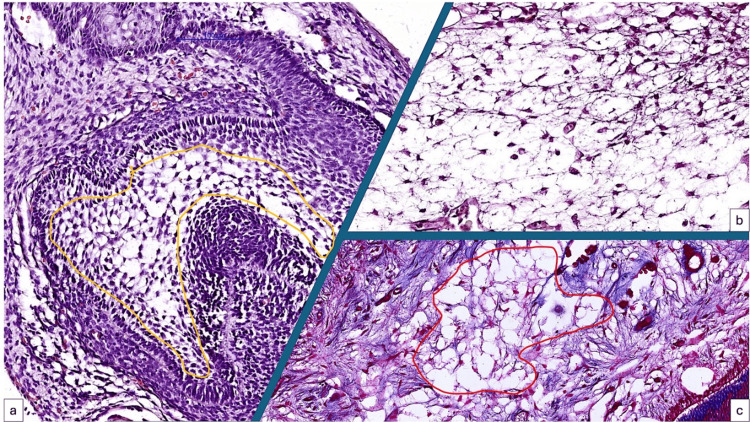
Histologic similarities between developing tooth enamel organ (a, yellow quadrant), the chorion of the CAM (b) and the inner part of the adult human tooth (c, red quadrant). Stellate-shaped interconnected cells are the microscopic pattern for all three structures. CAM: Chorioallantoic membrane

The CAM chorion cells mimic the microscopic structure of the enamel organ from the early stages of tooth development and mature tooth dental pulp cell architecture (Figures 2a-2c). Both the CAM chorion and tissue precursor for the enamel organ are composed of stellate-shaped mesenchymal cells with rapid potential for differentiation.

For both developing enamel organ and CAM, the mesenchymal cells are distributed between two epithelial tissues which potentiate the mesenchymal cell’s ability to differentiate into other mature cells. Moreover, these tissues contain as one of the main components of their extracellular matrix the hyaluronic acid and its derivatives [34-36]. Epithelial to mesenchymal induction during developing tooth and CAM plays an important role in the future common functional state of both tissues.

Human dental pulp stem cells induce a strong angiogenic response when they are implanted on the CAM and possess high migratory potential induced by hypoxia or neurodegenerative conditions.

Over 20 years ago, Gronthos et al. showed that the tooth pulp tissue had a population of stem cells that resembled mesenchymal stem cells [37]. The progenitor cells known as human dental pulp stem cells (hDPSCs) exhibit remarkable ability to differentiate into a variety of cell types in vitro, including odontoblasts [38], osteoblasts [37, 39,40], chondroblasts [37,39,40], adipocytes [40], neuronal cells [41,42], and hepatocyte-like cells [43]. One study reported that hDPSCs have also the ability to differentiate into endothelial cells [44], but their complete differentiation remained questionable as compared with other mesenchymal stem cell sources from the head and neck [44,45]. hDPSCs offer numerous benefits for clinical applications when compared with other adult mesenchymal stem cells, primarily due to their straightforward isolation from adult teeth. Moreover, these stem cells maintain their ability to differentiate into multiple lineages even after cryopreservation, thereby paving the way for the establishment of a stem cell bank [46].

The angiogenic potential of hDPSCs was mainly certified by using the CAM assay, through grafting alone or by using different biomaterials usually used in dental clinical practice [47] and were validated by other experimental models [48]. hDPSCs exert their angiogenic potential due to their phenotype characterized by the expression of VEGF, MCP-1, plasminogen activator inhibitor-1 (PAI-1), and endostatin, found both at the mRNA and protein levels [28]. Interestingly, they promote CAM neovascularization due to their ability to increase endothelial cell migration but not endothelial proliferation [28] and their angiogenic potential is lower than that of bone marrow-derived mesenchymal stem cells as it has been reported recently on the CAM assay [49]. Recently, Meng et al. improved the CAM assay on hDPSCs by combining this experimental model with molecular methods [50]. Cobalt chloride (CoCl2)-induced hypoxia hDPSCs increased neovascularization on the CAM and promoted angiogenesis through a paracrine mechanism and the inhibition of miR-143-5p upregulated the proangiogenic potential of hDPSCs under hypoxic conditions by directly targeting HIF-1α pathway [50]. By using the CAM, the hDPSC's ability to transmigrate through biological membranes has been reported to be higher than that of BM-MSCs in the context of neurodegenerative milieu [51].

Ameloblasts, odontoblasts, and their derivatives in the CAM assay

There are very limited data about ameloblasts, odontoblasts, and their derivatives used in the CAM assay. None of the published papers used human-derived ameloblasts and/or odontoblasts in the CAM assay with emphasis on the development and function of both ameloblasts and odontoblasts and their impact on neovascularization. All the studies performed on the CAM used animal-derived ameloblasts and odontoblasts harvested from the early stages of embryonic tooth development [11-13,32]. By contrast, with the lack of ameloblasts and odontoblasts used in the CAM assay, their derivatives were directly or indirectly tested on the CAM. EMDs promote angiogenesis in the CAM. EMDs have numerous physiological activities, although it is likely that at least some of the reported favorable clinical effects are due to angiogenesis stimulation [23]. EMD proteins seem to improve wound healing and have a role in the regenerative process of periodontal tissues [23,52]. Amin et al. [53] proved that enamel matrix protein has two different fractions with different functions on neovascularization and characterizes a tyrosine-rich amelogenin peptide (TRAP) fraction able to up-regulate the expression of the endothelial markers, including VEGF receptor 2 (VEGFR2), Tie-1, Tie-2, VE-cadherin and von Willebrand factor (vWF) and to induce neovascularization on the CAM [53].

Scaffolds, biomaterials, and dentistry-specific bone substitute evaluation on the CAM assay as a prerequisite for the future dental restorative material characterization

**Figure 3:**
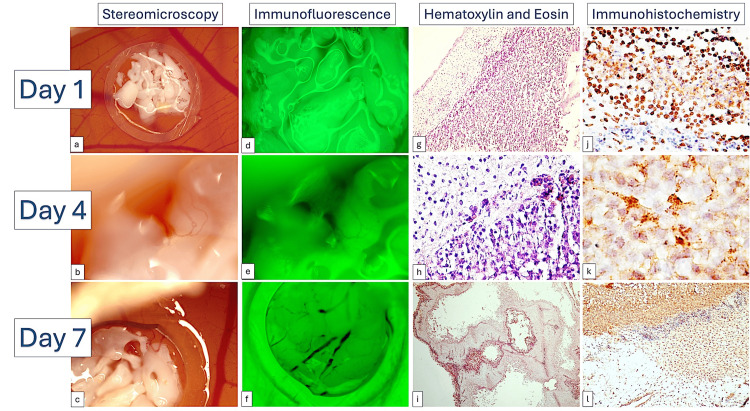
BioOss implant on the CAM assessed related to its ability to acquire blood vessels and to express osteoblastic markers. Dynamic evaluation of the BioOss implant mixed with hyaluronic acid from day 1 (a, d, g, j) to day 4 (b, e, h, k) and day 7 (c, f, i, l). Note the high ability of the implant to acquire vessels gradually (b, e, c, f) and to express RUNX2 (j), BMP4 (k), and SPARC (l) the known markers of osteoblastic differentiation. This is an original figure obtained by us during experimental research. CAM: Chorioallantoic membrane

Alveolar ridge augmentation serves as an initial phase in dental implant procedures. Numerous studies have been published concerning the techniques employed and biomaterials used for alveolar ridge augmentation. The primary issue in this field pertains to the characterization of biomaterials employed for augmentation and their impact on host tissues. Certain augmentation techniques are intricate and entail invasive procedures that are often poorly tolerated by patients. The CAM model enables the evaluation of the initial interactions between bone substitutes and mesenchymal cells involved in bone regeneration. The microscopic and molecular events associated with alveolar bone renewal remain poorly understood due to insufficient experimental models and a limited range of molecular markers for evaluating this process. Usually, the observations about bone substitutes or scaffolds evaluation on the CAM assay remained limited to the macroscopic observation. Intrinsic processes of osteoblastic differentiations are poorly described. In 2018, Cirligeriu et al. grafted several different combinations of hyaluronic acid mixed with a bone substitute used in dentistry and assessed not only the angiogenic potential of the implant but also the CAM's ability to induce osteoblastic differentiation [30]. As an in vivo substrate of cells with a high potential for differentiation, undifferentiated CAM mesenchymal cells are comparable to those found in alveolar bone. These cells can differentiate into the osteoblastic lineage. The authors reported that the differentiation of CAM mesenchymal cells toward the osteoprogenitor and osteoblastic lineages was induced by the bone substitute/hyaluronic acid (BH) complex. The BH complex is an optimal combination for alveolar ridge augmentation, as it does not induce inflammation and confirms its osteoinductive effect on mesenchymal cells through the RUNX2+, BMP4+, and SPARC+ phenotype, which is capable of bone matrix synthesis and mineralization (Figure 3).

Furthermore, this study endorses the use of mesenchymal cells in combination with bone substitutes and hyaluronic acid for the purpose of augmenting the alveolar ridge [30]. A more recent study confirmed the previously reported data and highlighted that hyaluronic acid added to bone substitutes significantly induced angiogenesis in vivo, leading to a faster integration and an improved healing in clinical settings [54]. A mixture of hydrogels containing hyaluronic acid, cellulose nanocrystals, and platelets lysate was tested on the CAM for its ability to induce angiogenesis but also to attract cells for dentin-pulp junction regeneration [55].

The regenerative dentistry research area has become increasingly interested in the development of dental bone substitutes that are scaffold-type dental ceramics with varying porosity, which are based on 3D scaffolding [56]. A quick and efficient method utilizing the CAM assay has been established to quantitatively characterize angiogenesis and vascular conduction in scaffolds. This method enhances conventional cell culture assays and may partially substitute in vivo investigations. It was used on silicon-substituted hydroxyapatite porous bioceramics featuring diverse pore geometries. The material was determined to be biocompatible, facilitating the conduction of blood vessels on its surface. The existence of pores does not affect angiogenesis; nevertheless, the geometry of the pores influences blood vessel orientation and angio-conductive capability. Pores having a triangle cross-section are especially appealing for the design of scaffolds to enhance vascular colonization, osteointegration, and overall performance [57].

Several combinations of hemostatic biomaterials proved their angiogenic ability when they were implanted in the CAM by accelerating wound healing due to a proper vascular support [58]. Combinations between various biomaterials and different cells with ability to differentiate into osteoblasts were tested on the CAM assays as part of their preclinical validation for their use in future clinical practice [59-65]. Together with well-known human mesenchymal stem cells, the investigators combined different biomaterials and scaffolds with cells originating from gingiva (progenitor cells or gingival fibroblasts) [63,66], dental pulp [29, 46-48] or bone marrow-derived mesenchymal stem cells [49].

Irritation tests performed for dental adhesives or dental alloys, cytotoxic activity assessment, and dental antibacterials/antiseptics activity evaluated using the CAM assay

Acidic fluorides be used to cure dental erosion. When tested on the CAM assay, the irritation capability of diluted hydrofluoric acid (HF) solutions for possible application in the oral cavity appears to differ. According to HET-CAM, 0.05% HF was mildly irritating, 0.1% HF was highly irritating, and 0.2% and 1% HF were extremely irritating [67]. Eugenol's possible toxicity was examined in ovo, and at the maximum quantity (1 mM) studied, it had a moderately irritating effect on the CAM [68]. Fluoride-containing dental products have been used extensively over the past few decades to prevent tooth decay and obtain good oral hygiene. Nonetheless, worries about the possible toxicological effects of fluoride exposure have spurred ongoing research. Only NaF produced irritating effects in ovo studies on the CAM, indicating a possible negative effect on the vascular system [69]. Due to Because of its broad-spectrum antibacterial activity, chlorhexidine (CHX) is one of the most often used antiseptics in dentistry. However, there is growing evidence about the possible harmful events that could result from its widespread use in practice. CHX digluconate doses of 0.02% and 0.2% exhibited the maximum cytotoxicity, along with an irritating impact on the CAM [17]. Due to their ability to improve the strength of implants and composite materials, promotion of cell adhesion and proliferation, and antibacterial properties, carbon nanotubes have attracted attention in the dental sector [70]. Research on the combination of magnetic nanoparticles and inorganic metals (like silver) has potential in the field of dentistry. Without affecting the embryo's short-term toxicity, Fe3O4@carbon nanoparticles caused a mild irritation sign restricted to a small location [70]. Dahl used the HET-CAM to evaluate 27 dental adhesive products that covered the four adhesive concepts: self-etch with one or two phases and etch and rinse with two or three steps. The test system was the blood vessels on the CAM and the degree of irritation was determined by evaluating how the blood vessels responded to the test chemical over the course of five minutes of exposure. Three distinct end-goals, blood coagulation, blood lyses, and blood vessel rupture, were assessed, and their corresponding appearance time points were recorded. The most common harm was blood coagulation, which occurred in 25 out of the 36 tested solutions within less than a minute of exposure. Of the solutions, 16 were classified as strong irritants and 17 as moderate irritants. Neither the presence of 2-hydroxylethyl methacrylate (2-HEMA) in the products nor the kind of solvent (water, ethanol, or acetone) could be associated with the type or intensity of the reaction [71].

CAM assay and dental organoids: a bridge from the present to the future in dental research

Tissue-derived organoids are created by embedding tissue cells or fragments in a three-dimensional extracellular matrix scaffold, usually Matrigel, and exposing them to a specific growth factor cocktail that resembles developmental signaling pathways and tissue stem cell niches. Compared to conventional 2D cell cultures, these organoid models closely mimic the phenotypic and functional traits of the original tissue. They are also extendable while maintaining their unique properties. After being exposed to differentiation factors or co-cultured with other cell types, these stem cell organoids can differentiate into tissue cell types. Hermans et al. [72] reported the establishment of organoid models derived from both mouse molars (MTO) and incisors (ITO). The derived epithelial organoids exhibit long-term expandability, replicate tooth-specific traits, and demonstrate differentiation capabilities of dental epithelium stem cells in both in vivo and in vitro settings. This is further supported by the inclusion of dental mesenchymal (pulp) stem cells, reflecting the critical epithelial-mesenchymal interactions that occur during tooth development. MTO and ITO were transferred on the CAM assay used as a platform for in vivo validation of in vitro established dental organoids. ITOs can survive, retain epithelial nature (CK14+), and produce both AMELX and ODAM (enamel matrix proteins), whereas TOs can survive and spontaneously differentiate toward ameloblast-like cells in vivo [72].

By testing several dental compounds usually used in clinical practice related to their toxicity, irritative ability or to induce bleeding we may select the low-toxic materials for clinical practice (Table 1).

**Table 1:** Chemical compounds and biomaterials used in dental clinical practice and tested on the CAM As shown in the table, an increased number of papers have been published in 2024 about CAM assay use in dental research CAM: Chorioallantoic membrane

Compound /Biomaterial	Year	Authors	CAM Assay Results	Clinical Impact	Ref.
Sodium alginate/aloe vera/sericin (SA/AV/S)	2024	Bhoopathy et al.	Angiogenic properties	Scaffold for hemostatic materials development	[58]
Sodium fluoride (NaF), Xylitol (Xyl	2024	Breban-Schwarzkopf et al.	NaF induced vascular lysis and microbleedings. Xyl induced a mild dilation of blood vessels	Potential therapeutic strategies in the realm of cytotoxicity studies.	[69]
Chlorhexidine digluconate	2024	Dinu et al.	Irritant effects independent of its concentration. Vascular lysis and blood coagulation	The exposure of healthy gingival and cutaneous cells to CHX digluconate results in concentration- and time-dependent cytotoxic events	[17]
Leukocyte and platelet-rich fibrin (L-PRF) and the modified low-speed advanced PRF (A-PRF)	2024	Nair et al.	Exposure to both L-PRF and A-PRF increased the angiogenic potential.	Periodontic wound healing improvement	[73]
Acellular dermal matrix (Epiflex)	2024	Sagheb et al.	Revascularization ability and angiogenic properties	Soft tissue augmentation in dentistry and implantology	[74]
Fibrin-alginate-calcium phosphate biomaterial (FACaP)	2024	Owji et al.	Bone cell differentiation, biocompatibility tests, vascularization	Bone augmentation in dentistry	[75]
An oxygen (O_2_)-releasing hyaluronic acid (HA)-based dispersion with a controlled oxygen delivery	2023	Müller-Heupt et al.	Increased angiogenesis, lack of cytotoxic effects	Tissue regeneration improvement in periodontitis based on O2 release	[76]
Iodixanol	2017	Hertig et al.	Neutral related to angiogenesis, cell toxicity, or irritation	Radioopaque fibrin hydrogel formulation for endodontic treatments	[77]
Polymeric scaffold with Bromelain and magnesium-doped hydroxyapatite nanoparticle	2020	Shoba et al.	Ability to acquire blood vessels and to sustain a well-defined vascular network	Periodontal regeneration	[78]
Low concentration of Hydrofluoric Acid (HF)	2009	Hjortsjö et al.	0.05% HF was slightly irritant, 0.1% HF moderately irritant, 0.2% and 1% HF strongly irritant	Accidental exposure of soft tissues to solutions containing more than 0.2% HF may be harmful.	[67]
"Classic" alloy compositions	2005	Ardlin et al.	1 mmol l(-1) Cu(2+) solution was graded as slightly irritating	Safety of alloy use for patient's health	[79]

**Figure 4:**
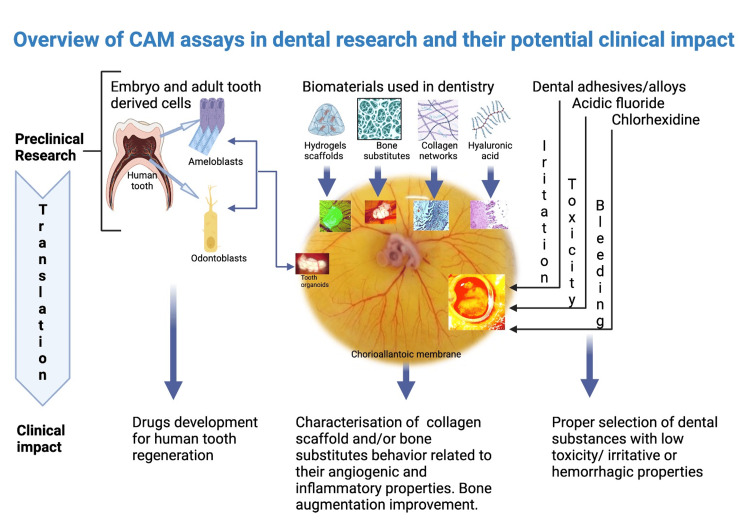
Overview of CAM assays in dental research and their potential clinical impact. Preclinical dental research results can be translated into clinical practice. From induced enamel development to irritation and cytotoxicity tests, CAM assay is a reliable preclinical platform in dental research. This is an original figure created by the authors themselves. Figure 4 is created using special software (https://www.biorender.com/) dedicated to the graphical collage which may be inserted in the research articles. This service provides originality certificate for each created picture and also it gives us permission for publication. CAM: Chorioallantoic membrane

The CAM model is a versatile experimental tool for preclinical studies in the field of dental research. Its highly vascular support gives it the ability to adapt to heterogeneous material implanted on it and also to integrate and to be integrated in most of the biomaterials tested on it. The first tooth organoid grown on CAM starting from mouse dental stem cells is already available and provides strong insights for the development of human tooth organoids. Several drugs and growth factors may be tested by combining them with such dental organoids. Vascularization and inflammation play a pivotal role in the integration of dental implants but also in the periodontal disease. Bone augmentation is a prerequisite step for a successful future implant so, bio-scaffolds need to be vascularized, with no inflammation but also with the ability to differentiate into osteoblastic lineage. Graphical summarization of CAM assays is presented in Figure 4 together with their potential impact on dental clinical practice.

Alternatives for chick CAM assay in dental research**

As an alternative to chick CAM assay, two other species must be considered for future use in dental research: ostrich and alligator CAMs. The ostrich CAM has a big surface area and gives the opportunity to co-culture several biomaterials to study their cross-interactions but also with dental tissues harvested from human and animal specimens. The ostrich CAM model has a double incubation time compared to the chick CAM and it gives us the opportunity to perform long-term experiments especially those involving dental pulp mesenchymal stem cell differentiation, enamel and/or dentine formation, especially for tooth organoid implants or dental human and animal embryonic tissues derived from the primitive oral cavity. Due to its higher thickness and special vascular network development [80], some hard biomaterials could be implanted on the ostrich CAM and can be dynamically evaluated in close relationship with dental tissues. Even entire dental lamina obtained from embryonic mouth development could be entirely implanted on the ostrich CAM for studies related to the early steps of teeth development. At this moment, no dental research has been developed by using the ostrich CAM, this being used only as a platform for breast cancer tumor xenografts [81]. Thus, we consider that the ostrich CAM may be implemented in dental research as a validation method for the studies developed on chick CAM. 

Crocodilians demonstrate continuous tooth replacement and are recognized as appropriate models for tooth regeneration studies owing to the anatomical similarities in dental cavities and teeth between these animals and humans [33]. The alligator CAM comes quickly behind with a more extended period of incubation (63 to 68 days). The alligator CAM model could be an alternative for studying cells or factors involved in the natural teeth regeneration known to be present for this species. Recently, researchers paid much attention to alligator CAM research and proved that it is highly resistant to hypoxia compared to other CAMs [82]. This comes in response to previous studies developed on Nile crocodile embryos related to the manipulation of in ovo embryonic dental tissues [33]. These results will be beneficial for forthcoming embryo-based investigations concerning jaw and tooth development in crocodiles, as well as research on human tooth regeneration.

## Conclusions

The CAM experimental model respects the 3R rules of the Research Ethics. Because it does not induce pain and suffering in the embryo, it will be considered in the future as one of the most recommended experimental models in research. Compared to other animal models, the highly vascularized CAM is an accessible and affordable way to screen for angiogenesis but it is also proper for performing other tests as toxicological or developmental tests. Despite several advantages given by the CAM model, this experimental tool was unfortunately less used in dental research. Its similarities to dental tissues, the ability to mimic the mesenchymal background needed for tooth development, the lack of an inflammatory microenvironment, and its cell ability to differentiate into several types of dental tissue cells highly recommend the CAM as a reliable experimental tool for dental research. 
